# MDM2 inhibitors-mediated disruption of mitochondrial metabolism: A novel therapeutic strategy for retinoblastoma

**DOI:** 10.3389/fonc.2022.1000677

**Published:** 2022-10-20

**Authors:** Arianna Romani, Enrico Zauli, Giorgio Zauli, Saleh AlMesfer, Samar Al-Swailem, Rebecca Voltan

**Affiliations:** ^1^ Department of Environmental and Prevention Sciences and Laboratorio per le Tecnologie delle Terapie Avanzate (LTTA) Centre, University of Ferrara, Ferrara, Italy; ^2^ Department of Translational Medicine, University of Ferrara, Ferrara, Italy; ^3^ Research Department, King Khaled Eye Specialist Hospital, Riyadh, Saudi Arabia

**Keywords:** retinoblastoma, retina, MDM2 inhibitors, Nutlin-3, mitochondrial metabolism, p53

## Abstract

MDM2 is the principal inhibitor of p53, and MDM2 inhibitors can disrupt the physical interaction between MDM2 and p53. The half-life of p53 is very short in normal cells and tissues, and an uncontrolled increase in p53 levels has potential harmful effects. It has been shown that p53 is frequently mutated in most cancers; however, p53 mutations are rare in retinoblastoma. Therefore, therapeutic strategies aimed at increasing the expression levels of wild-type p53 are attractive. In this minireview, we discuss the potential use of nutlin-3, the prototype small molecule inhibitor that disrupts the MDM2-p53 interaction, for the treatment of retinoblastoma. Although p53 has pleiotropic biological effects, the functions of p53 depend on its sub-cellular localization. In the nucleus, p53 induces the transcription of a vast array of genes, while in mitochondria, p53 regulates mitochondrial metabolism. This review also discusses the relative contribution of p53-mediated gene transcription and mitochondrial perturbation for retinoblastoma treatment.

## Introduction

Retinoblastoma is the most common intraocular pediatric cancer that arise in the retina. In developing countries that harbor approximately 80% of patients with retinoblastoma, the mortality rate is about 60% (1). Worldwide, the standard incidence rate of retinoblastoma varies from 1.8 (in native Americans) to 6.0 (in Southeast Asian population) per million person-years in children aged 0-14 years, occurring as a bilateral or unilateral disease (2, 3). Bilateral tumors are mainly due to new germline mutations, whereas unilateral tumors are linked to somatic mutations in the developing retina (3). In countries with a good health care system, the survival rate of children with retinoblastoma is high; however, patients at an advanced stage have a high risk of metastasis, vision loss, and globe rupture (4). For early diagnosed tumors, chemotherapy (combination of vincristine, carboplatin, and etoposide (VCE) as standard of care or topotecan, anthracyclines, and melphalan as alternative therapies) in association with adjunctive local treatment increases globe survival in approximately (90%) of cases when the treatment is promptly initiated (4, 5), while plaque radiotherapy is used as the last option (6). Enucleation is the most common approach for preventing metastasis in patients with advanced retinoblastoma (1). Although retinoblastoma etiopathogenesis has been debated for a long time, recent studies have demonstrated that retinoblastoma arises from post-mitotic cone precursors during the development (7–9). The proliferation of these precursors is dependent on E2F-regulated genes and these cells strongly express both *MYCN* and *MDM2* (8, 9). In 95% of the cases, tumor arises due to the biallelic loss of the tumor suppressor gene *RB1*, and its development is sustained by genetic and epigenetic factors that lead to leukocoria, a white pupillary reflex (3).

*RB1* and *TP53* are the main tumor suppressor genes that regulate pathways involved in the cellular response to insults (10). Rb and p53 interact to regulate the cell cycle *via* the p53-p21-Rb axis, in which p21, a cyclin-dependent kinase inhibitor, plays a pivotal role in regulating Rb phosphorylation (11). Loss or inactivation of *RB1*, as observed in retinoblastoma, leads to the sustained activation of E2F family proteins resulting in uncontrolled cell proliferation. Interestingly, unlike most cancers, mutations in *TP53* are rare in retinoblastoma (3). Nevertheless, during retinoblastoma progression, upregulation of MDM2/4 prevents p53-mediated apoptosis, thus promoting tumor survival and favoring uncontrolled cell proliferation by reducing basal levels of p53 (10, 12). Under normal physiological conditions, MDM2, an E3 ubiquitin ligase, ubiquitinates p53 leading to its subsequent degradation *via* the 26S proteasome, thus maintaining the p53 levels low.

MDM2 plays important roles in various cancers. It has been shown that MDM2 overexpression confers resistance to conventional chemotherapy (13, 14). Thus, pharmacological and/or genetic interventions that could restore or reactivate the p53 pathway by reducing the p53-MDM2 interaction, is a rational approach that was demonstrated *in vitro* as well as *in vivo* in xenograft models with significant tumor regression (15–17). This review focuses on MDM2 inhibitors as p53 reactivating agents for retinoblastoma treatment. Moreover, the review also describes the roles of p53 in different cellular compartments: nuclear p53 as a transcription factor and mitochondrial p53 as a regulator of mitochondrial metabolism.

## MDM2 inhibitors

Several molecules have been developed that can inhibit the MDM2-p53 interaction. Nutlins (nutlin-1, -2, and -3) are the first synthetic molecules that act as potent and selective MDM2 inhibitors (18). It has been demonstrated that the active enantiomer nutlin-3a (IC50 ~90 nM) possesses anti-tumor activity *in vitro* (19). Nutlins are *cis*-imidazoline analogs that interact with the p53 binding pocket of MDM2 (20). Nutlin-3a exerts a vast array of biological effects, such as cell cycle arrest, enhancement of senescence, and induction of apoptosis by stabilizing p53 (21). However, the pharmacological properties of nutlins are suboptimal for clinical application, thus, their use is limited to preclinical studies (20).

The first clinically tested molecule is RG7112 (IC50 ~20 nM), a new member of the nutlin family (20, 22, 23). Furthermore, idasanutlin, a second-generation molecule (RG7388, IC50 ~6 nM), possesses better pharmacokinetic properties, enhanced potency, better selectivity, and enhanced bioavailability compared to the first-generation nutlins and RG7112. Idasanutlin can be orally administrated, is relatively well-tolerated, and dose-dependently stabilizes the p53 protein (23, 24). Recently, idasanutlin monotherapy and combination therapy have been proposed, and a phase III clinical study on acute myeloid leukemia (AML) has been recently terminated (25–27).

The imidazopyrrolidinone siremadlin is an advanced molecule that has been tested in patients with solid tumors and hematological malignancies harboring no p53 mutations. In a phase I clinical study involving patients with AML, siremadlin has demonstrated promising results (28). Moreover, siremadlin is being investigated alone or in combination with other drugs in five different clinical trials that are still recruiting patients (29–34). Other MDM2-targeting molecules that have entered phase I clinical trials for solid tumors or hematological malignancies include ALRN-6924 (35), milademetan (36), AMG-232 (37, 38), CGM097 (39), APG-115 (40) and BI-907828 (41, 42). Of these, only the dual MDM2/MDM4 inhibitor, ALRN-6924 in combination with cytarabine is under investigation in retinoblastoma (43). This study is ongoing since 2018 and still recruiting patients; however, no results have been published yet.

## Action of MDM2 inhibitors on nuclear gene transcription and mitochondrial metabolism

**Figure 1:**
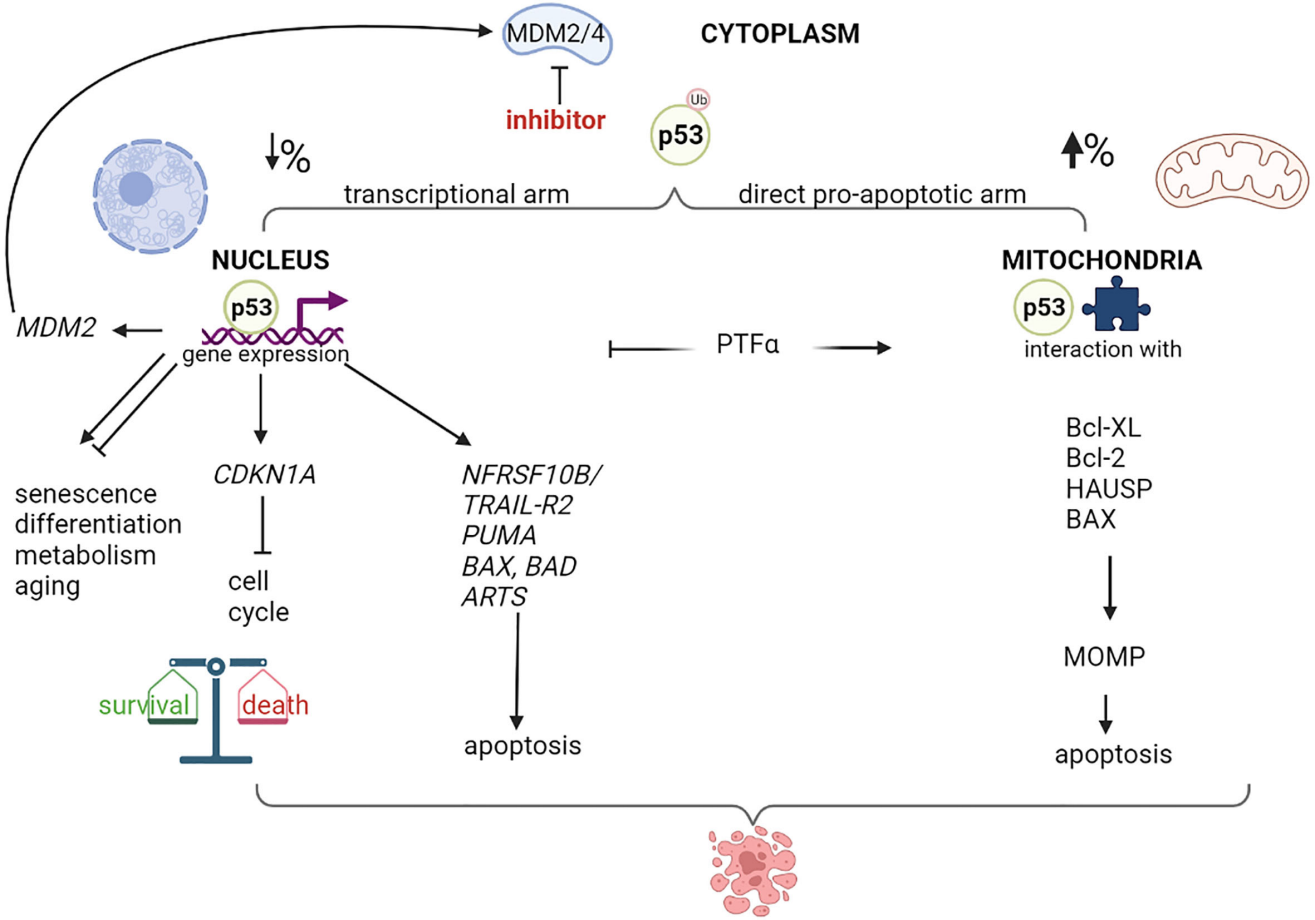
Nuclear and mitochondrial p53 destination after MDM2/4 inhibition. Schematic representation of different destination of p53 after treatment with a MDM2/4 inhibitor. Cytoplasmatic monoubiquitinated p53 can move through nucleus or mitochondria and activate programs of gene expression or direct apoptosis induction, with different percentage depending on cellular context. p53-mediated expression modulates live and death pathways, resulting typically in cell cycle block and apoptosis after MDM2 inhibition. A negative-feedback control is represented by the *MDM2* p53-dependent expression. Treatment with a specific inhibitor of p53-mediated transcription, such as Pifithrin-alpha (PTFα), orients the program to mitochondria, resulting in mitochondrial damage, membrane permeability (MOMP) and higher apoptotic levels. Created with Biorender.com .

Nutlin-3a has been shown to induce apoptosis in different types of cancer cells, including retinoblastoma; however, the relative role of the p53 transcriptional activity or mitochondrial functions, remains unclear (5, 44–46). Following nutlin-3 treatment, p53 levels increase rapidly, and p53 is either translocated to the nucleus, where it regulates transcription, or localized to mitochondria (**Figure 1**). It has been hypothesized that the indirect effects of MDM2 inhibitors on the nuclear transcription machinery might contribute to their cytotoxicity by inducing the transcription of pro-apoptotic genes, such as *NFRSF10B/TRAIL-R2* (47, 48), *PUMA* (48–51), *BAX* (51–54) and the apoptosis-related protein in TGF-β signaling pathway *ARTS* (55). However, several studies have shown that inhibition of p53 transcriptional activity (by other agents) significantly increases MDM2 inhibitors-mediated cytotoxicity (44, 56–58). A possible explanation for these paradoxical findings could be that p53 simultaneously activates the pro-apoptotic and pro-survival pathways (59–61). The balance between these opposing effects highly depends on the cell type and relative nuclear p53 levels. Increasing the nuclear p53 levels depletes the pool of cytoplasmic p53, which is more effective in promoting apoptosis by acting on the mitochondria (62). Steele et al. showed that following nutlin-3a treatment, a major fraction of p53 remains stably associated with the mitochondria where it binds to Bcl-2 (56). Moreover, pifithrin-α, an inhibitor of p53-mediated transcription, blocked the up-regulation of *PUMA* levels. Surprisingly, pifithrin-α dramatically augments apoptosis induced by molecules that increase intracellular p53 levels. Another study showed that mono-ubiquitinated p53 preferably translocates to the mitochondria in response to stress (44). Of note, nutlin-3 does not interfere with the ability of MDM2 to monoubiquitinate p53 because MDM2-p53 complexes are only partially disrupted by nutlin-3 treatment, and nutlin-3-stabilized MDM2 retains its E3 ubiquitin ligase activity. Blocking the transcriptional arm of p53 using α-amanitin or pifithrin-α greatly potentiates the nutlin-induced apoptosis (44, 56). In summary, the direct mitochondrial program is a major mechanism in nutlin-induced p53-mediated apoptosis.

At the mitochondrial molecular level, nutlin-3 reduces the levels of dihydrolipoamide dehydrogenase/dihydrolipoamide acetyltransferase protein complexes, which leads to the disruption of the pyruvate dehydrogenase complex and inhibition of the mitochondrial activity (58). Moreover, nutlin-3 along with bortezomib induces dilatation of the endoplasmic reticulum (ER) and mitochondria (63). Interestingly, it has been shown that a negative feedback loop exists at the mitochondrial level which prevents p53-induced apoptosis (64). Nutlin-3-induced mitochondrial translocation of p53 stimulates ERK1/2 activation, an anti-apoptotic signal, *via* mitochondrial ROS generation.

When p53 is monoubiquitinated by MDM2, such as in the presence of nutlin-3, is stabilized (44). This modification promotes p53 translocation to mitochondria, where p53 is deubiquitinated by herpesvirus-associated ubiquitin-specific protease (HAUSP), and the deubiquitinated protein enhances the mitochondrial outer membrane permeabilization by interacting with Bcl-2 family proteins (BclXL/Bcl2 and Bax) (65). Both *in vitro* as well as *in vivo* studies have shown that p53 induces apoptosis by altering the mitochondrial outer membrane permeabilization, although the p53 protein lacks a mitochondrial localization signal. It is widely accepted that ubiquitination patterns drive the fate of proteins. For instance, polyubiquitination of proteins at Lys48 acts as a cellular signal for proteasomal degradation, whereas multi-(lysine)-monoubiquitinated proteins are stable in the cytosol and this modification acts as a signal for intracellular trafficking (65, 66). Moreover, the second generation MDM2 inhibitor idasanutlin has been reported to enhance the phosphorylation and degradation of Mcl-1, promote Bak release and mitochondrial membrane damage, and induce apoptosis (52).

In parallel, MDM2 inhibitors can directly affect mitochondrial bioenergetics independently of p53 due to the role of MDM2 in integrating respiration and apoptosis (67–69). Cytosolic MDM2 can translocate to the mitochondria and suppress the transcription of NADH-dehydrogenase 6 (*MT-ND6*), which is present in the mitochondrial genome, thus, inhibiting respiration and inducing ROS (53). Localization of MDM2 to mitochondria is accompanied by ultrastructural changes in the organelle, such as reduction in matrix electron density and misoriented and reduced cristae, and these changes are not associated with increased apoptosis (53). Interestingly, this type of mitochondrial morphology is observed in retinoblastoma (described in the next section), which leads to the hypothesis that a large fraction of MDM2 could localize to the mitochondria and induce changes in its morphology and functions. These specific features of MDM2 can enhance the p53-mediated effects of MDM2 inhibitors and help induce cytotoxicity in cancer cells, even in the presence of mutated or altered p53.

## Mitochondrial morphology and metabolism in retinoblastoma

**Figure 2:**
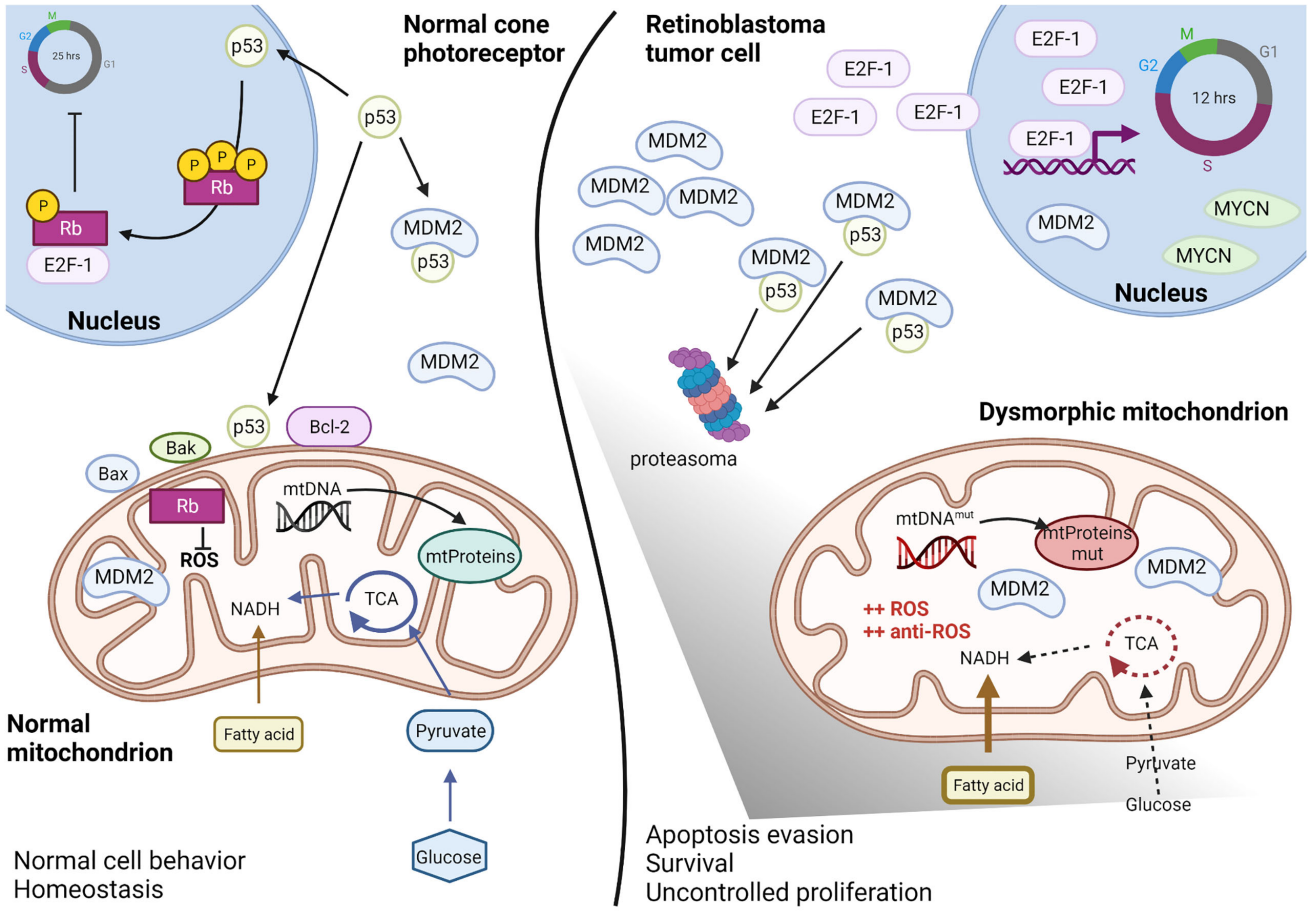
Altered mitochondria in retinoblastoma. Schematic representation of the altered mitochondria and cellular environment in retinoblastoma tumoral cell respect to a normal cone photoreceptor, as proposed by recent literature data discussed in paragraph 4. In the normal cell (left), basal levels of p53 can regulated both nuclear and mitochondrial effects and maintain the intracellular homeostasis. The presence of Rb protein guarantees both the cell cycle inhibition as well as the ROS control. In the retinoblastoma tumoral cell (right), Rb is lost and high levels of MDM2, E2F-1 and MYCN favorite p53 degradation and uncontrolled cell cycling. Mitochondria metabolism is deregulated with predominance of fatty acids instead of glucose consume, DNA mutations in the D-loop region and ROS hyper-production compensated by anti-ROS hyper-induction. Therefore, mitochondria appear dysmorphic and aberrant but functional for survival and apoptosis escape. Created with Biorender.com.

Several common ocular diseases, such as glaucoma and diabetic retinopathy are characterized by mitochondrial dysfunction in the neural retina and retinal pigment epithelium (RPE). For such diseases, recent studies have focused on improving mitochondrial functions to restore vision, with therapeutic approaches directed to ameliorate mitochondrial membrane potential and stability, ROS production, mitochondrial fusion and fission, mitochondrial biogenesis, mitophagy, apoptosis, and mitochondrial DNA (mtDNA) transcription (70). Retinoblastoma is not classified as a mitochondrial disease; however, evidence suggests that mitochondria are affected in this disease (**Figure 2**). In normal retinal cone cells, mitochondria exhibit a specific distribution and organization. These organelles are present in the inner segments parallel to the orientation of the cell that helps in concentrating light onto the outer segment of the photoreceptors (71, 72). Since the original morphology of the cone cell is lost in the tumor, the mitochondria appear disorganized in the cytoplasm of the retinoblastoma cells. Moreover, ultrastructural analyses of poorly differentiated retinoblastoma revealed reduced mitochondrial number and morphological aberrations. These mitochondria appear swollen or elongated due to fusion-fission phenomena, and partial or complete alteration of cristae is evident, with membrane loss in some mitochondria (73). Some of these features, particularly aberrations in cristae with partial or complete cristolysis, have also been observed in retinal cell organoids and are associated with the tumor phenotype (7).

Clearly, altered mitochondrial structure in retinoblastoma cells indicates some degree of mitochondrial dysfunction. Furthermore, Nicolay et al. reported that in *RB1^KO^* RPE cells, mitochondrial respiration associated with proton leakage is reduced during the electron transfer (74). Defective electron transfer to oxygen increases the production of superoxide ions, which act as a growth signal (75). Simultaneously, free reactive species promote lipid peroxidation and tumor invasiveness to the choroid, optic nerves, and orbit (76). Despite the increase in ROS production, the oxidative balance is maintained, preventing cell death. Indeed, it has been shown that levels of the antioxidant enzymes, such as superoxide dismutase, catalase, and glutathione peroxidase, are increased in Y79 cells following peroxide stimulation (77). Moreover, in the serum of retinoblastoma patients, the GSH/GSSG ratio is reportedly high, suggesting the abundance of reduced form, which acts as a cellular antioxidant (78). Conversely, high levels of the mitochondrial-associated protein LRPPRC inhibit autophagy, and in Y79 and WERI-Rb-1 cells, high levels of LRPPRC activate ROS production and promote tumor progression (79).

In addition, Singh et al. published a detailed investigation of the mtDNA, analyzing the mutations in the displacement loop (D-loop) region in 60 Rb tumors (80). The authors demonstrated that high-frequency mutations in the mtDNA D-loop correlate with altered mitochondrial structure, as described previously, indicating the possible role of these mutations in the etiopathogenesis of retinoblastoma.

Other mitochondrial functions that are altered in retinoblastoma include energy metabolism. In a recent comparison of retinoblastoma tumors to pediatric retina, it has been observed that advanced retinoblastoma have lower expression of glycolytic genes, particularly *HK1*, and altered expression of Krebs cycle-related genes, suggesting reduced dependence on glycolysis and altered Krebs cycle, together with a preference for fatty acid metabolism when compared to the average of all pediatric retinal cells (81). Moreover, in the same comparison, these tumors exhibit lower expression of FOXO3, a protein that regulates apoptosis and autophagy under normal conditions. FOXO3 triggers apoptosis in the absence of survival factors, and low expression of FOXO3 in advanced retinoblastoma indicates the tumor strategy to escape apoptosis. However, further studies are required for a detailed understanding of how differences between retinoblastoma cells versus total pediatric retina relates to retinoblastoma tumorigenesis.

In summary, although mitochondria are dysmorphic in retinoblastoma cells, their functions are maintained to ensure tumor cell proliferation. At the molecular level, rapid proliferation of the retinoblastoma cells is due to the loss of Rb, which leads to sustained and high levels of free E2F family proteins. Simultaneously, p53 levels are maintained low in these cells due to high MDM2 and MDM4 expression.

## Preclinical studies of MDM2 inhibitors in retinoblastoma

Despite encouraging *in vitro* results of nutlin-3 in retinoblastoma cell lines and primary cells with *TP53* ^wild-type^/*RB1* ^mutated^ background (10, 46, 82), results of the *in vivo* experiments are not very promising. Animal models receiving systemic intravenous and oral nutlin-3 administration failed to achieve IC_50_ in the vitreous and retina due to the low permeability of the blood-ocular barrier (83).

To overcome this limitation, local delivery of nutlin-3a through subconjunctival injection has been performed in an orthotopic retinoblastoma xenograft model; however, the reduction in tumor volume was low, but the results were significantly improved with topotecan combination (10). Brennan et al. developed an ocular formulation of nutlin-3a (nutlin-3a^oc^) for subconjunctival administration, and using this formulation, 2000- and 20000-fold higher intravitreal nutlin-3a levels can be achieved compared to oral and intravenous administration (45). This formulation has been validated in combination with systemic topotecan in two mouse models of retinoblastoma: MDMX mice (constitutively expressing MDMX, *Chx10-Cre; Rb^lox/lox^; p107^-/-^; MDMX^Tg^* background) and p53TKO mice (*Chx10-Cre; Rb^lox/lox^; p107^-/-^; p53^lox/lox^* background). Results showed that the activation of p53 was higher and the response was overall better in mice constitutively expressing MDMX than in mice lacking p53, confirming that nutlin-3a activates p53 in cells with high MDMX levels (45). In an orthotopic and more aggressive model of retinoblastoma, subconjunctival nutlin-3a^oc^ administration combined with systemic topotecan, showed significant improvement in survival due to tumor necrosis and activation of the p53 pathway compared to that observed using multimodal chemotherapeutic regimens (VCE or carboplatin/topotecan) (45)

## Discussion and future perspectives

Clinical applications of MDM2 inhibitors are limited due to drug resistance as a result of mutations in MDM2, p53, or other proteins involved in the response pathways, or due to the off-target effects (22). To reduce the selection pressure, innovative therapeutic approaches are needed, such as MDM-2 inhibitors in combination with other drugs and genetic tools targeting different molecular pathways or targeting/activating other cell types present within the tumor microenvironment.

Indeed, new synergistic therapies for retinoblastoma have recently been developed by combining DNA-damaging drugs (topotecan and etoposide) with inhibitors of DNA repair agents (B02), and this combination has synergic effects on p53-dependent cell death leading to a reduction in Y79 or RB1021 cell-derived tumors, but not in WERI-Rb1 cell-derived tumors in xenograft models (84). This drug resistance of WERI-Rb1 cell-derived tumors is due to the preference for p21 rather than Bax following p53 activation, which can be prevented by using navitoclax, a Bcl2/Bcl-XL inhibitor. Navitoclax/topotecan, navitoclax/B02, or navitoclax/topotecan/B02 combination act synergistically to promote apoptosis in WERI-Rb1 cells by inducing Bax activation in the mitochondria *via* the p53 pathway (84). These results represent another proof of concept that the activation of the p53 pathway is beneficial in retinoblastoma and support the possibility that MDM2 inhibitors, the non-genotoxic activators of p53, may be used in combination with other drugs targeting intracellular pathways to overcome drug resistance. Another strategy for retinoblastoma treatment could be MDM2 inhibitors in combination with gene therapy using the oncolytic adenovirus CVN-01, which selectively replicates in tumor cells with high levels of free E2F-1. CVN-01 has been used successfully in primary retinoblastoma cells, xenografts, and in a preliminary phase I trial that reported a reduction in vitreous tumor seeds in one patient (85). Another approach could be modifying the tumor microenvironment by targeting angiogenesis. In retinal vasculature, nutlin-3 possesses anti-neoangiogenic activity and does not affect mature blood vessels (86). However, the issue of drug resistance while using a single agent remains a constrain, and combination with known antiangiogenic drugs, such as bevacizumab, could attenuate the selective pressure leading to the inhibition of angiogenesis and tumor cell death, as observed in a xenograft model of neuroblastoma (87). Moreover, nanoparticles, such as trans-ethosomes, have recently been proposed as ophthalmic formulations (88), with local noninvasive methods of treatment.

Recently, retinal organoids have been generated from patient-specific induced pluripotent stem cells (iPSC), which may help in further advancement of the preclinical research (7, 89, 90). These organoids represent a unique tool for investigating novel therapeutics and their impact on the differentiation, proliferation, morphology, and metabolism of tumors. Retinal organoids can be used to study the effects of MDM2 inhibitors on mitochondria within the original tumor since these organoids have a 3D architecture that permits full cell-to-cell contact. Indeed, in the normal retina, the plasma membrane contact between the adjacent inner segment of photoreceptors, together with the correct alignment of the mitochondria inside the cell, seems fundamental for mitochondrial functions, photoreceptor homeostasis, and correct visual capacity (71). Since retinoblastoma cells have alterations in the number, localization, and morphology of mitochondria (73), retinal organoids can be used to study the effects of MDM2 inhibitors, either alone or in combination, on p53-dependent mitochondrial metabolism in a close-to-real setting, including that observed during the early phases of tumor development.

We believe that the development of new preclinical models together with recent results from clinical trials, may open new opportunities for the treatment of retinoblastoma and encourage the use of MDM2 inhibitors.

## Author contributions

Conceptualization, GZ. Writing original draft preparation, GZ, RV, and AR. Writing review and editing, GZ, AR, EZ, SA, SA-S and RV. All authors contributed to the article and approved the submitted version.

## Funding

This work received no external funding. Funds for open access publication fees were received by RV as local funding from University of Ferrara (FIRD program).

## Acknowledgments

We would like to thank Editage (http://www.editage.com) for editing and reviewing this manuscript for English language.

## Conflict of interest

The authors declare that the research was conducted in the absence of any commercial or financial relationships that could be construed as a potential conflict of interest.

## Publisher’s note

All claims expressed in this article are solely those of the authors and do not necessarily represent those of their affiliated organizations, or those of the publisher, the editors and the reviewers. Any product that may be evaluated in this article, or claim that may be made by its manufacturer, is not guaranteed or endorsed by the publisher.
